# A Decentralized Architecture for Trusted Dataset Sharing Using Smart Contracts and Distributed Storage

**DOI:** 10.3390/s22239118

**Published:** 2022-11-24

**Authors:** Miguel Pincheira, Elena Donini, Massimo Vecchio, Salil Kanhere

**Affiliations:** 1 Digital Industry Center, Fondazione Bruno Kessler, 38123 Trento, Italy; 2 Digital Society Center, Fondazione Bruno Kessler, 38123 Trento, Italy; 3 School of Computer Science and Engineering, University of New South Wales (UNSW), Sydney, NSW 2052, Australia

**Keywords:** blockchain, decentralized file systems, smart contracts, data sharing, IPFS, swarm

## Abstract

The data economy is based on data and information sharing and tremendously impacts society as it facilitates innovative collaborations and decision-making strategies. Nonetheless, most dataset-sharing solutions rely on a centralized authority that rules data ownership, availability, and accessibility. Recent works have explored the integration of distributed storage and blockchain to enhance decentralization, data access, and smart contracts for automating the interactions between actors and data. However, current solutions propose a smart contract design limiting the system’s scalability in terms of actors and shared datasets. Furthermore, little is known about the performance of these architectures when using distributed storage instead of centralized storage approaches. This paper proposes a scalable architecture called DeBlock for data sharing in a trusted way among unreliable actors. The architecture integrates a public blockchain that provides a transparent record of datasets and interactions, with a distributed storage for data storage in a completely decentralized way. Furthermore, the architecture provides a smart-contract design for a transparent catalog of datasets, actors, and interactions with efficient search and retrieval capabilities. To assess the system’s feasibility, robustness, and scalability, we implement a prototype using the Ethereum blockchain and leveraging two decentralized storage protocols, Swarm and IPFS. We evaluate the performance of our proposed system in different scenarios (e.g., varying the amount and size of the shared datasets). Our results demonstrate that our proposal outperforms benchmarks in gas consumption, latency, and resource requirements, especially when increasing the number of actors and shared datasets.

## 1. Introduction

Recently, the European Commission highlighted the tremendous impact on our society of the data economy [1]. Sharing data and information facilitates innovative collaborations in research and company ecosystems and contributes to decision-making strategies impacting society. Over the last decade, the fast progress in information and telecommunications technology allowed the availability of data and information at an unprecedented scale [2].

Existing data-sharing platforms (e.g., open data portals and data marketplaces) generally rely on a central authority regulating the users and data, acting as a third-party intermediary. The intermediary enables trust among the participants and manages the system by defining the policies for data ownership, access, and usage and by setting the rules to evaluate data reliability and integrity [2]. However, intermediaries favor usability over transparency as they aim to connect data owners with users rather than to facilitate the availability of data to anyone [3]. Hence, the central authority controls data access and usage and can discriminate among different users. Moreover, a centralized system suffers from a single point of failure, which is a well-known problem in cloud services [4]. The breakdown of the central node has several negative consequences, including data breaches, denial of service, and data loss [5]. Therefore, a need emerges for a system that enables sharing and retrieving data without needing a trusted third party (TTP).

Blockchain can address this need as it guarantees trusted interactions among the users without needing the TTP. Blockchain provides a platform that combines cryptography, data structures, and incentive mechanisms to maintain a unique and immutable record of information in a peer-to-peer network [6]. Originally introduced for cryptocurrency [6], the inherent decentralization provided by blockchain has had a great impact in several other scientific fields, including edge computing [7], AI [8], resource management [9], and Internet of Things [10]. Furthermore, blockchain technology is intrinsically verifiable and immutable, as all the actors in the network can access the information without being able to modify anything. For these reasons, blockchain is a suitable technology for sharing data among untrusted actors without needing a TTP [11]. Nevertheless, blockchain does not support storing a large amount of data given the different constraints imposed by various practical issues (e.g., block size, transaction fees, and latency) [6,12]. Consequently, data sharing requires blockchain to be integrated with centralized or decentralized off-chain services to store the data. Traditional centralized cloud-storage services, such as google drive, dropbox, or even Github, remove blockchain’s decentralization benefits by introducing an intermediary (i.e., the service provider). Conversely, decentralized storage, also called Decentralized File Systems (DFS), does not require the TTP and preserves the decentralization introduced by blockchain. Two of the most common DFS protocols are InterPlanetary File System (IPFS), and Swarm [13]. However, there has been little quantitative analysis of the architectures for integrating blockchain and DFS, particularly in terms of scalability, robustness, and performance in data-sharing applications.

### 1.1. Related Works

An increasing number of works are integrating blockchain and distributed storage for applications in several domains, such as Industry [14], Healthcare [15], and IoT [16], to name a few. Furthermore, a recent survey by Ismail et al. [17] highlighted the importance of decentralized storage systems, not only as a complement to blockchain applications but as an alternative to centralized storage services. The authors surveyed nine DFS (e.g., Filecoin [18], Sia [19], and Arweave [20]) providing long-term decentralized storage, where a blockchain incentivizes the users and pays for the storage space. Hence, their focus differs from sharing datasets between untrusted actors.

Other solutions [21,22,23,24,25,26,27] propose the association of a private blockchain with a DFS network to provide a decentralization of the dataset storage. Some solutions store the datasets in the DFS platform, and the link to the datasets in the blockchain [23,24,25,26,27]. Other approaches propose to store on the blockchain information related to the authors or the shared dataset. For example, the authors in [22] store the dataset metadata to increase traceability. Authors in [21] store the information to cryptographically check the integrity and availability of a file in the blockchain. However, private blockchains require a central authority that assumes different forms, such as a group of actors called approves [27] to add and assign a cryptography signature for every new peer [26]. This may reduce the decentralization and openness introduced by the blockchain as the central authority should approve each participant (or user).

Some studies, e.g., [16,28,29,30], explored the integration of public blockchain and DFS networks to maintain the decentralization properties and open the system to any untrusted users.

To automatize the data access and retrieval, interactions between the actors and the network are controlled by smart contracts, whose design critically influences the system performance in terms of time and cost (GAS). For example, the authors in [16] propose a smart contract for each kind of dataset structure to handle the retrieval. However, this reduces the scalability, and the system’s generalization as dataset structure not considered in the system design cannot be shared. The authors in [29] propose to generate a smart contract for each actor willing to share data and for each data query, making the architecture hardly scalable to many actors. Moreover, a smart contract for each actor implies a degradation of the performance in terms of cost and time with the increasing number of actors in the system. Moreover, each dataset query requires a transaction that modifies the blockchain’s state, significantly increasing the computational load and thus the cost which is one of the main barriers to adopting these types of solutions [17]. Although both approaches use a public blockchain to guarantee decentralization and automation, they lack a quantitative evaluation of the system performance in terms of time and cost. However, the evaluation is critical to understanding the robustness and scalability of the system when varying the number of actors and shared datasets. Further, they are mostly designed for specific use cases, such as IoT big data [16], and do not generalize to all types of data, e.g., images and text. Finally, none of the approaches tracks the dataset evolution over time, e.g., the updates and integration. For example, considering IoT sensors that continuously acquire measurements, data can be shared every few months by updating the already shared dataset. Summarizing, a need emerges for an architecture for data sharing that (i) is completely decentralized both in managing and storing data, (ii) ensures trusted interactions among unknown actors, (iii) ensures dataset traceability, (iv) ensures the data integrity, accessibility, and auditability, and (v) is scalable in terms of actors and shared datasets. Moreover, an evaluation of the performance is needed to understand the system’s robustness, accessibility, and scalability.

### 1.2. Novel Contribution

This paper proposes a novel architecture to share trusted data between untrusted actors without needing a third-party authority as an extension of the work in [31]. The architecture relies on a public blockchain that is open to anyone and uses a DFS network for storage. The architecture exploits the blockchain to store the information on the dataset (i.e., metadata) and the DFS network to store the dataset, creating a fully decentralized solution. The interactions between the users and the network for the data upload and retrieval are governed by smart contracts. We propose two types of smart contracts: the catalog smart contract tracks the data in the system, and the dataset smart contract allows fast and efficient retrieval. This software architecture guarantees a scalable system regarding the number of actors and shared datasets, with time responses comparable to those offered by a centralized cloud system without including an intermediary.

We implement a proof of concept of DeBlock using the Ethereum blockchain network and two different DFS networks– InterPlanetary File System (IPFS) and Swarm. Then, we evaluate the system performance in different scenarios (e.g., varying the amount and size of the shared datasets), considering as metrics the transaction cost and processing time, the data upload and download time and the node resources’ impact in terms of memory and CPU impact. We also benchmarked against a baseline to demonstrate the scalability and efficiency of DeBlock when increasing the number of actors and shared datasets.

In summary, the main contributions of this paper are: (i) the use of a public blockchain acting as a central authority, which guarantees trust among untrusted users and dataset access, integrity, and traceability; (ii) the design of two types of smart contracts, i.e., catalog and dataset, that guarantee the scalability of the system in terms of actors and shared datasets; and (iii) the evaluation of the proposed architecture performance to assess the scalability and robustness with respect to literature architectures and centralized storage.

### 1.3. Structure of the Paper

The rest of this paper is structured as follows. Section 2 introduces blockchain technology and the distributed storage system. Then, Section 3 proposes the architecture focusing on the interactions between the users and the network. Section 4 reports the architecture set-up and experiments to evaluate the proposed architecture performances. Finally, Section 5 concludes the paper and presents the future work.

## 2. Blockchain and Decentralized Storage

### 2.1. Blockchain Technology

This Section briefly describes blockchain technology and the unique features used in our proposed architecture. Blockchain is a technology that enables trust without intermediaries by providing a transparent and immutable list of records. The technology merges data structures, incentive mechanisms, and cryptography techniques to create and maintain a special distributed database on a peer-to-peer network [10,31]. The first use case for this technology is cryptocurrencies (e.g., Bitcoin [6]), where a blockchain system stores financial transactions between unknown parties, acting as a distributed Ledger. Nonetheless, several other use cases emerged in the last few years [9,10], confirming that systems can be adopted in other applications where data exchange occurs between untrusted actors.

The three main components of a blockchain system are the transactions, the blocks, and the network state. Transactions store information about the information exchange between two system actors. A block is the data structure that groups transactions with additional cryptographic safeguards and is validated by all the peers. Finally, the information in the validated blocks creates a unique global state that all the network peers agree upon [31].

#### 2.1.1. Blockchain Protocol

**Figure 1:**
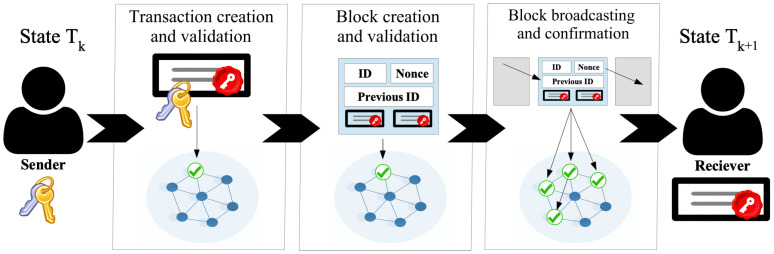
Block scheme of the blockchain protocol for creating and validating a transaction, creating and validating a block, and broadcasting the block in the network from an agreed state in $T_{k}$ to an agreed state in $T_{k+1}$.

A blockchain system is governed by a protocol that describes (i) how to create and validate transactions, (ii) how to create and validate new blocks, and (iii) how to broadcast the block and update the state of the network [31]. This protocol applies whenever two peers interact, as shown in Figure 1 and described in the following subsections.

Transaction creation and validation. The identity of each actor in a blockchain is represented by a unique address associated with a pair of cryptographic keys. Actors use these keys (i) to sign their transactions to certify their origin and (ii) to validate the integrity and the origin of the transaction they receive. Blocks collect valid transactions and are distributed in a peer-to-peer network and thus are accessible to any actor. Hence, Blockchain is a transparent and verifiable record of interactions (property of auditability) [10].

Block creation and validation. A block is a time-stamped data structure, grouping transactions and linked to the previous block. When creating a block, the protocol applies cryptographic techniques to validate it, using a hashing function to create a unique identifier (i.e., the block ID). This ID protects the block (and the content) from tampering since it loses its validity with any data change in the block’s content. Furthermore, the link with the previous block creates a retroactive relation that contributes to securing the Blockchain: any modification implies heavy computations to validate and seal the previous and following blocks. Thus, the information in Blockchain is considered permanent in time (property of immutability) [31].

Block broadcasting. Finally, when a new block is broadcast to the network, each peer appends it to the local copy of the chain after validating it. Therefore, each peer has a copy of all the blocks in the Blockchain, providing a distributed architecture to the system, and thus, it is tolerant to data failures. Appending the block implies its validation and the update of the Blockchain global state that is agreed upon by all the peers, following a consensus algorithm. The consensus dictates how to resolve conflicts, avoid abuses related to personal interests over the common good, and incentivize participants without intermediaries. In this way, interactions occur directly among peers without any central control. Consequently, the consensus protocol makes the system decentralized, which is an intrinsic property of Blockchain [31,32].

#### 2.1.2. Blockchain Taxonomy

There are two main types of Blockchain: permissionless (also known as public) and permissioned (also known as private) networks. A permissionless blockchain, such as Bitcoin [6], Ethereum [33], and Litecoin [32], is open, and anyone can join the network and create new blocks. Permissionless blockchains rely on unknown users to operate as network peers in exchange for cryptocurrency incentives as the processing fee for validating transactions [34]. Malicious behaviors in the block validation are discouraged by the cryptocurrency amount [35] or the computational resources for solving a cryptographic puzzle [6] needed when creating new blocks. In a permissioned blockchain, an organization or a group controls access to the network to a limited number of peers and defines different roles and permissions for the users. New blocks are published by authorized nodes, reducing the security constraints and thus, increasing the performance of the system [32] without processing fees for the transactions. Even if a private blockchain provides auditability and offers better performance (e.g., lower latency, higher transaction throughput), it is not entirely decentralized or censorship-resistant as a public blockchain. Moreover, a private blockchain is not as tamper-resistant as a public blockchain as the organization may roll back the Blockchain to any point in the past.

#### 2.1.3. Scripting Capabilities and Smart Contracts

The central protocol supports additional features that can improve the functionalities of a blockchain system. One of the most relevant features is the smart contracts [36]. Smart contracts are software stored in the Blockchain, originally developed to take advantage of blockchain features to implement and enforce agreements between two or more parties in an autonomous way [37]. Opposite to Bitcoin, which gives limited scripting capabilities, Ethereum provides a Turing-complete language to build software that runs on top of the Blockchain, using the peers as a distributed computer. The software runs deterministically in all the peers simultaneously to process the information in the Blockchain. The exact execution output enforces the agreement among peers without the need for any third-party validator. Smart contracts have been the key to the expansion of Blockchain to other domains beyond cryptocurrencies. While Bitcoin is considered the reference implementation for the blockchain protocol, the reference for smart contracts is Ethereum [38]. Permissionless blockchains have taken Ethereum as the model for implementing smart contract functionalities.

### 2.2. Distributed Storage

**Figure 2:**
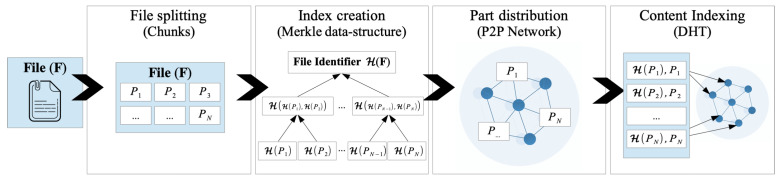
Block scheme of the decentralized storage protocol for splitting a file F into *N* pieces $P_{n}$ stored along with the hash $H(P_{n})$ in the peers.

Distributed Storage, also called Distributed Files Systems (DFS), provides a decentralized infrastructure to store data in multiple nodes, typically over a peer-to-peer network, in a replicated fashion [13]. DFS overcomes several challenges of centralized cloud storage, such as data reliability, availability, and integrity. The underlying peer-to-peer network enables an efficient auto-scaling system without a single point of failure, creating a highly reliable storage infrastructure [13]. Furthermore, since a DFS simultaneously stores the files in several locations, the content is censorship-resistant with higher availability despite individual failures of particular nodes [13]. Regarding security and privacy, cryptographic data structures provide embedded tamper-proof of the content, creating an additional layer of integrity verification. Two of the main DFS protocols are the InterPlanetary File System (IPFS) and Swarm [13]. Both protocols provide distributed storage with a content delivery protocol but with differences in design and implementation in terms of the network layer, the peer management protocol, and the data structure used. IPFS is more mature in terms of development and adoption [39]. Swarm is developed on the Ethereum protocol and thus fully integrated with the smart contracts [40]. Although IPFS and Swarm have different protocols [13,39], they share several similarities in both design and implementation, see Figure 2.

The simplified protocol to upload and store a file in a distributed storage network is depicted in see Figure 2 and described in the next paragraph. When uploading a file F, the DFS divides it into *N* smaller pieces $P_{n}$ so that $F=\{P_{1},\ldots ,P_{N}\}$. Each $P_{n}$ is processed by a cryptographic hashing function H(·) that generates a unique cryptographic hash. For each $P_{n}$, the hash $H(P_{n})$ acts as unique identifier. A Merkle data structure (Merkle Tree for Swarm and Merkle DAG for IPFS, respectively) connects all the hashes of all the pieces at different levels [41]. At the bottom level of the tree, the hashes $H(P_{n})$ and $H(P_{n+1})$ of pieces $P_{n}$ and $P_{n+1}$ are connected to generate $H(H\left(P_{n}\right),H\left(P_{n+1}\right))$. Finally, at the root of the tree, a unique hash for each file H(F) is generated (see Figure 2). The use of Merkle data structures [41] creates a unique identifier for the file based on its content. Merkle data structures provide beneficial properties for content addressing, optimizing disk usage, and file integrity. Each piece of file $P_{n}$ is stored on different network peers along with the corresponding hash $H(P_{n+1})$. Using the hashes in a Distributed Hash Table provides an efficient routing mechanism to address uploaded files and their pieces among the network peers. In addition, if any $P_{n}$ is corrupted or tampered with, the hash changes for the entire file, enabling quick integrity verification.

## 3. Proposed Architecture

This work proposes a fully decentralized architecture called DeBlock to share and retrieve datasets in a trusted and traceable way without an intermediary. The architecture integrates a permissionless blockchain and a decentralized file system (DFS). The blockchain network provides an immutable and transparent record to all the users (actors or network peers) and thus, guarantees traceability and trust. The decentralized file system network guarantees dataset availability and integrity as they are encrypted and always available. Hence, DeBlock guarantees the integrity, ownership, and availability of the dataset on the network to all the actors in a trusted and decentralized way. DeBlock is based on three main assumptions that are easily satisfied. The actors are (i) identified via the cryptographic public/private keys, which are securely stored, (ii) have legal rights over the shared data, and (iii) the encryption operations are securely performed outside the blockchain (off-chain).

Actors. We assume a group of $X=\{X_{i},i\in \left[1,\ldots ,N_{X}\right]\}$ actors that are willing to share and retrieve data. Since the actors are unknown to each other, they are untrustable. Therefore, the datasets to share and retrieve cannot be trusted, which means that the datasets’ integrity, availability, and accessibility cannot be guaranteed. The actors sharing the datasets are called data-owners and are the owners of the intellectual property of the group of datasets $Y=\{Y_{j},j\in \left[1,\ldots ,N_{Y}\right]\}$. We assume that data owners are willing to share the data, but in a secure and traceable way to preserve the ownership and the intellectual property. In the context of scientific research, they also want recognition and acknowledgment of the dataset used. The actors willing to retrieve datasets are called data users, and they need a way to verify the dataset’s origin, integrity, and changes, i.e., evolution over time. Normally, this task is done by an intermediary that connects and mediates between two users—here, the blockchain network acts as an intermediary, enabling trusted and direct interactions between unknown actors. Note that an actor can act both as a dataset owner and data user within different interactions.

Metadata and Dataset. Here, we assume that each data $Y_{j},j\in \left[1,\ldots ,N_{Y}\right]$ consists of the metadata $M_{j},j\in \left[1,\ldots ,N_{Y}\right]$ and the dataset $D_{j},j\in \left[1,\ldots ,N_{Y}\right]$. The metadata $M_{j}$ collects all the information describing the data, e.g., the data owner, acquisition time, and the data source. The numbers and types of metadata fields depend on the data type. $D_{j}$ is of the dataset to share that can have a different structure, e.g., a 1D signal measured by IoT sensors, or a 2D matrix, such as a satellite image.

### 3.1. Proposed Architecture Structure

**Figure 3:**
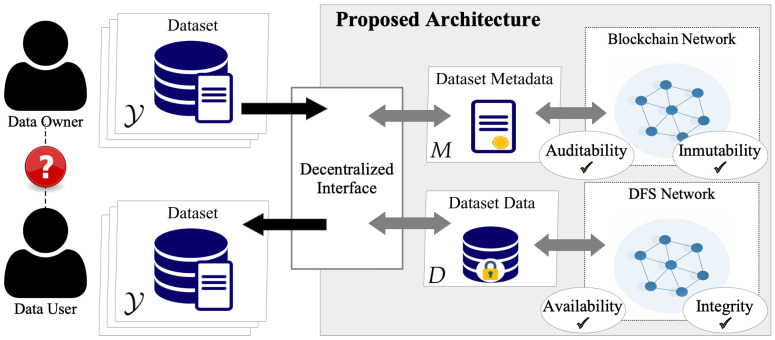
Block scheme of the proposed system architecture that provides an infrastructure for sharing datasets among untrusted actors.

The proposed architecture consists of an interface that connects two networks (see Figure 3), i.e., a public blockchain and a distributed file system (DFS). The blockchain network stores the metadata and keeps track of all the actor interactions with each dataset. For each dataset, the blockchain network records the ownership and the evolution (i.e., updates and changes), the identification (ID) of actors downloading it, and the evaluation. Evaluations are given by actors that previously downloaded the dataset. They can provide a score of the dataset goodness, such as the structure and quality. To increase the system’s openness, we propose integrating a permissionless blockchain that is open to any untrusted actor. The advantages of using a public blockchain in terms of transparency, availability, and openness are greater than the disadvantages, including costs, latency, and transaction throughput. A public blockchain enables any data owner to use the infrastructure as a trustless platform for directly interacting with unknown dataset users. As blockchain storage capacity is limited [10], datasets are stored in a DFS network to avoid any centralization. DFS increases the system liveness as the dataset is stored across multiple network peers. DFS provides each dataset with a unique identifier based on the dataset content. The ID is securely linked to the dataset owner to reduce possible mismanagement. Moreover, DFS has faster download and upload times than a centralized storing platform because of the multiple nodes storing the data. Finally, the decentralized interface (DI) acts as a coordinator, providing an interface to the blockchain and the decentralized storage networks. The decentralized interface interacts with the smart contracts and performs off-chain tasks, such as encryption and decryption of datasets. Each actor stores a DI copy to enhance the decentralization.

### 3.2. Proposed Architecture Interactions

**Figure 4:**
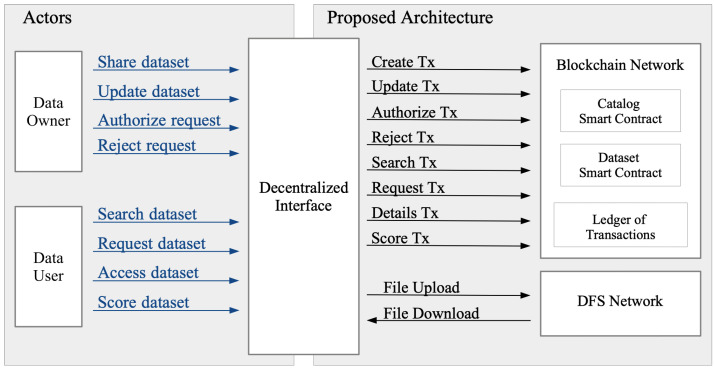
Modular view of the proposed system with the focus on the actors and their interactions.

Figure 4 shows the interactions between the actors and the proposed architecture, mainly depending on the actor type, i.e., data-owner or data-user. Data owners have three possible interactions: share, update, and authorize. Data users have three possible interactions: search, request, and score.

**Figure 5:**
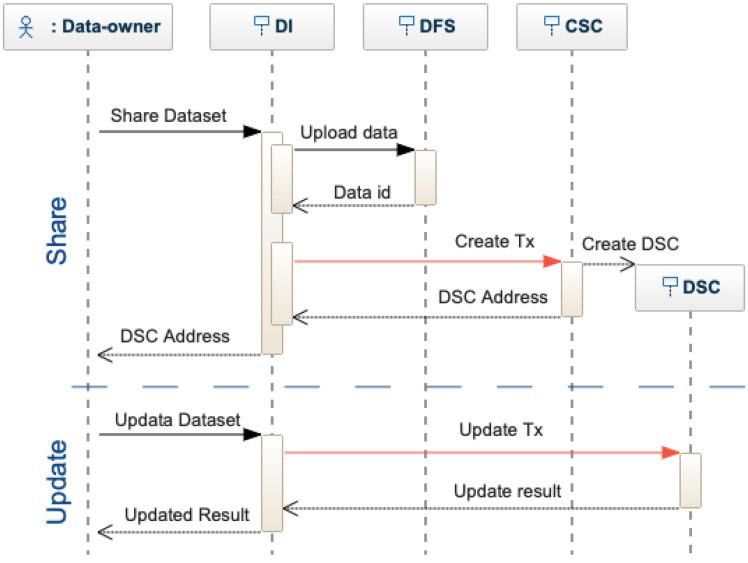
Sequence diagram for sharing and updating a dataset.

Dataset Owner. A dataset owner shares a dataset, i.e., metadata and the data, using the decentralized interface (see Figure 5). The dataset is encrypted and stored in the DFS, which provides a unique identifier (i.e., a cryptographic hash of the file). The identifier is included in the metadata and sent to the Catalog Smart Contract (CSC). CSC inserts the metadata in the dataset list and creates a Dataset Smart Contract (DSC). The DSC can be updated only by the dataset owner following a similar procedure for sharing. When a dataset is updated, a new identifier is generated by the DFS. The new and the old ID are stored on the blockchain, proving a traceable history of changes and modifications.

**Figure 6:**
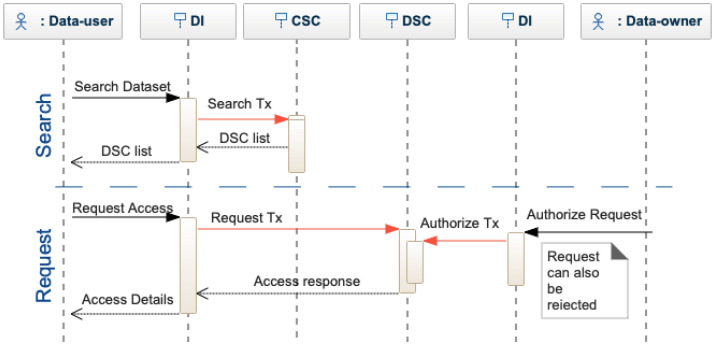
Sequence diagram for dataset search and access request.

**Figure 7:**
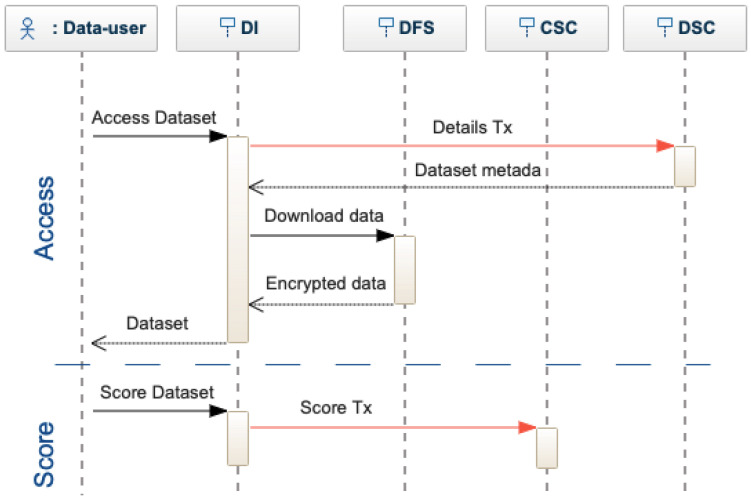
Sequence diagram for accessing and scoring a dataset.

Dataset User. A data user can search in the decentralized interface by using specific criteria, see Figure 6. The interface sends a search transaction to the CSC, which returns the list of matches. The actor can decide which dataset to use and require the complete metadata, querying each DSC individually. When finding a matching dataset, the data-user requests access to the data. The access request is transformed into a request transaction and sent to the DSC. The data owner of a dataset is notified of each access request that can be accepted or rejected. To authorize the request, the dataset owner provides the access key for the data, which is encrypted by the decentralized interface and sent to the DSC as an authorized request transaction. If the owner does not provide the keys, the request is rejected. After receiving access to a dataset, the actor is asked to evaluate the dataset quality (see Figure 7), providing a score. The actor receives a positive or negative reward. Based on the rewards, smart contracts can limit or encourage actor activities. Note that DeBlock supports the seamless integration of more complex rewarding mechanisms [13,16,31,42]. However, this falls beyond the scope of this paper.

### 3.3. Smart Contracts

Smart contracts define rules and methods to validate and process the actor interactions with the network and between them, providing an interface to access information. Smart contract design is critical as it is strongly linked with the system performance in terms of time and cost for uploading and retrieving data. Moreover, smart contracts are important for increasing system scalability, interoperability, and usability. To have fast and secure interactions between the users and the network, we propose two types of smart contracts, i.e., Catalog and Dataset.

#### 3.3.1. Catalog Smart Contract

The catalog Smart Contract (CSC) stores a list of the existing datasets and their quality scores, and thus provides user-friendly data retrieval capabilities to the architecture. Moreover, it indicates how to share, search, and score the datasets,

Dataset Sharing. CSC receives a transaction with the metadata coded in a standard format (e.g., ISO format). CSC first verifies the metadata format, then adds a new record in the dataset list, and finally rewards the actor. Adding a new record requires creating a Dataset Smart Contract (DSC) with a unique blockchain address stored in the dataset list (Algorithm 1).

Dataset Searching. The CSC receives a transaction with search criteria to filter the dataset list. The results contain the dataset identifiers matching the search criteria and the related quality scores.

Dataset Scoring. After downloading a dataset, an actor must send a transaction scoring the dataset quality to the CSC. Actors receive a positive or negative reward, depending on if they sent the evaluation or not. The reward sum indicates the reliability of an actor. 

**Algorithm 1:** Create new dataset in the CSC**  Input**: *dataset, owner, dataid***  Output**: *new_dsc.address*
  /* Verify that dataset does not exists    */
  **if**
*dataset in darasets_list*
**then**  ⌊ terminate  /* Create a new DSC                */  *new_dsc* = createDSC(*dataset, owner, dataid*)  /* Append new dsc to exists contract      */  datasets_list.append(*new_dsc*)  /* Reward the owner for sharing a dataset  */   owners_rewards[*owner*]++  return *new_dsc.address*

#### 3.3.2. Dataset Smart Contract

For each dataset, a smart contract (DSC) is created and owned by the actor that shared the dataset. Blockchain grants trust, while smart contracts provide easy access and autonomous interactions among actors. Furthermore, the DSC performs the basic validations of the user identity and permission, following the cryptographic rules of the blockchain. Nonetheless, the contract could also implement more complex validations of the dataset according to the capabilities of the blockchain platform (i.e., Turing completeness). Therefore, the DSC implements the methods to update, request, access, and authorize or reject the dataset access.

Dataset Updating. DSC processes transactions with the dataset changes from the owner. In addition, the DSC tracks all the changes that are accessible by anyone in the system.

Dataset Access. Accessing the dataset requires sending a transaction to the DSC. The DCS implements the logic for automatically giving access to the dataset or filtering requests, for instance, based on the requester score (see Algorithm 2).

**Algorithm 2:** Request access to dataset in a DSC

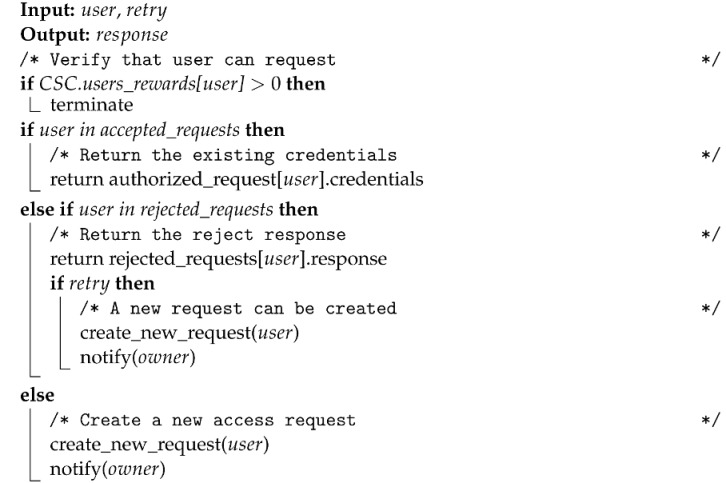



Dataset Request. Any dataset access request needs to be authorized by the dataset owner. The dataset owner provides the access key encrypted using the requester’s public key for the authorization.

The aforementioned smart contract design is innovative compared to those in the literature and has remarkable contributions that increase the system performance. The smart contract design improves the modularity of the system as (i) a DSD is created for any dataset, (ii) each DSD represents the interest and actions of the data owner, and (iii) one or more CSCs track all the datasets in the system. The CSC may be easily extended to (i) monetize the data, or (ii) incensing data-sharing and data-scoring with tokens.

### 3.4. Proposed Architecture Benefits

DeBlock presents several benefits derived from blockchain and distributed storage with a scalable smart contracts design. As described in Section 2, a blockchain system embeds auditability, immutability, and decentralization. Combining these features enables trust among peers based on an agreed global state of the network, removing the need for an intermediary. Similarly, DFS overcomes several challenges of centralized cloud storage, such as data reliability, availability, and integrity.

Therefore, in our architecture, any network peers can validate the integrity and origin of the dataset using the property of auditability. Moreover, instead of blind trust in a central authority, the cryptographic techniques provide the tools to perform the validation in a decentralized way. Moreover, these same cryptographic techniques guarantee the immunatibilty of the blockchain content. Thus, actors have full access to the complete history of the data, including the origin and following updates, without censorship or arbitrary edits. Furthermore, the consensus algorithm provides a system’s global status, agreed upon by the peers in a decentralized way.

Using a DFS, our architecture removes the intermediary for off-chain storage, rendering the system fully decentralized. The peer-to-peer network of DFS enables an efficient auto-scaling system without a single point of failure, increasing the reliability and availability of the stored datasets. Furthermore, the cryptographic data structures on DFS provide embedded tamper-proof of the content, creating an additional layer of integrity verification, transparent for the actors in the system.

Finally, smart contracts can automate task execution by encoding rules for ownership, formats, and rewards. The proposed smart contract design allows the system to scale easily in terms of actor and shared dataset, reducing the costs of transactions without sacrificing performance.

## 4. Experimental Setup and Results

This section evaluates the proposed architecture’s performance, potentialities, and limitations. First, we describe the experimental setup, including the hardware and software setup, parameters, and metrics used for the evaluation. Then, we present our evaluation divided into three objectives (i) managing the data, (ii) managing the metadata, and (iii) a brief security analysis.

### 4.1. Experimental Setup

#### 4.1.1. Hardware and Software Setup

Our architecture is blockchain agnostic, meaning it can rely on any blockchain network with scripting capabilities. For this evaluation, we tailored the blockchain module to the open-source project Ethereum [12] as it incorporates smart contracts as a Turing-complete language for software running on the blockchain [33]. To implement the Ethereum blockchain, we used the official Geth client (version 1.9.18-stable). To evaluate how different consensus algorithms might impact the architecture, we ran two independent nodes with different consensus algorithms, i.e., Proof-of-Work (PoW) and Proof-of-Stake (PoS). One node implements the Ropsten test network with PoW. The other node implements the Goerli network based on a variation of PoS. To reduce the security risks derived from smart contract vulnerabilities, we used industry-approved libraries in our implementation. For example, rewarding mechanisms and other critical functionalities are based on OpenZepellin, a library for a secure smart contract development considered a community-standard [38]. We used two identical virtual machines on an OpenStack server with 4 GB of Ram, 20 GB of SSD, and 4 vCPU, and running clean Linux Ubuntu installation (version 18.04).

For the decentralized storage module, we choose Swarm and IPFS as the most common DFS implementations [13]. IPFS [39] is more mature in terms of development and adoption. Swarm is integrated with the Ethereum protocol [40] and smart contracts. A cryptographic hash obtained from the content of the uploaded file uniquely identifies each document on both IPFS and Swarm. IPFS uses a multi-hash allowing different hashing functions, with SHA-256 as the default algorithm, and Swarm uses a hash function based on the SHA-3 algorithm. For a complete comparison of IPFS and DFS, we refer to [13]. As software tools for the decentralized storage module, we ran two independent nodes, using the official IPFS client (version 0.5.1) and the official Swarm client (version 0.5.7-5ccfd995). In addition, we used two identical virtual machines on an OpenStack server, with 4 GB of Ram, 20 GB of SSD disk, and 4 vCPU, running a clean Linux Ubuntu installation (version 18.04). The client module was implemented using Python (version 3.6) and ran on a Lenovo T490s notebook, with 16 GB of Ram 256 SSD disk, and an Intel i-7 processor at 1.90 GHz over a clean Linux Ubuntu (version 18.04).

#### 4.1.2. Dataset and Metadata Parameters

For the data stored on the decentralized file system, we considered seven sizes of files: 1, 5, 10, 50, 100, 250, and 500 MB based on the file sizes in literature [43]. Each file is a packed Unix archive (tar), including a single random bytes file and a text file with a unique timestamp and description for each experiment. Since the metadata depend on each use case, we considered raw data ranging from 100 to 1000 bytes based on the size reported in similar works [29]. Finally, the score dataset transaction is based on an ERC20 Token transfer transaction. The ERC20 is an Ethereum standard ensuring exchangeability and interoperability for all the blockchain tokens. The same token standard is used for rewarding the users. In both cases, the ERC20 token represents a unique and traceable unit of information (i.e., the score, the reward), and since it is a token, it can only be generated by authorized contracts in the architecture. Furthermore, this standard allows other actors outside the system to add additional value to this information, by making it exchangeable with other ERC20 tokens [9].

### 4.2. Experimental Results

#### 4.2.1. Performance of the Distributed Storage

**Figure 8:**
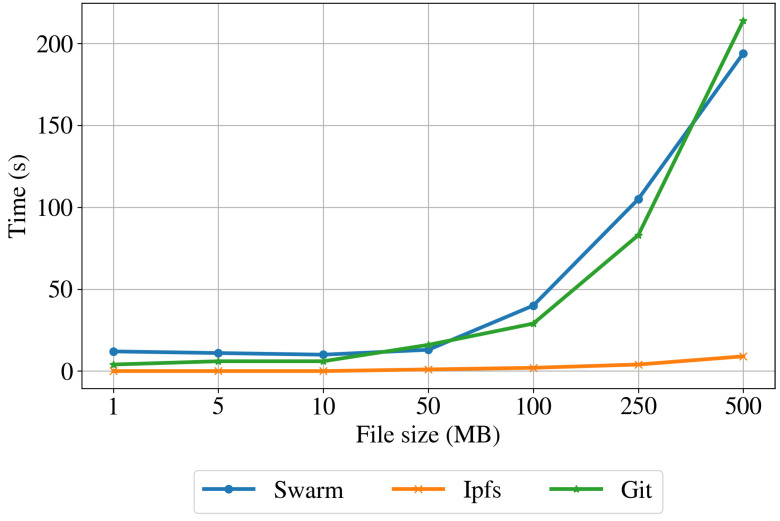
Average upload times for different file sizes for the Swarm, IPFS, and Git storage.

Dataset upload times. To evaluate the upload time in the distributed storage, we measured the upload times of the datasets as it is the main metric currently used when evaluating centralized architectures [29]. We created and uploaded ten different files of each size to evaluate the robustness and scalability of the system. In addition, we uploaded one file approximately every 30 min to evaluate different network conditions. To get a reference time compared to a centralized alternative, we also uploaded the files to a Git repository, pushing a new commit, and we used a shell script to measure the elapsed time. Table 1 shows the minimum, maximum, average, and standard deviation of the uploading time for the Swarm, IPFS, and Git implementations. In addition, the table includes the average peers connected to each node and the average ping time to the Git repository. These results are also plotted in Figure 8. The uploading time is always increasing for the centralized implementation (Git), showing an exponential increase. For the distributed implementations (Swarm and IPFS), the uploading time is almost constant for files smaller than 10 MB and increases for larger files. The distributed storage implementation based on Swarm has higher uploading times than the centralized implementation for files smaller than 375 MB. Conversely, the uploading time for files larger than about 375 MB is shorter than for the Git-based implementation. The IPSF implementation based has significantly lower uploading times than the Git and Swarm implementations for files of all dimensions. This is because the IPSF normally splits the data into fewer pieces and sends them to closer nodes, while the Swarm protocol privileges more split and farther nodes.

**Figure 9:**
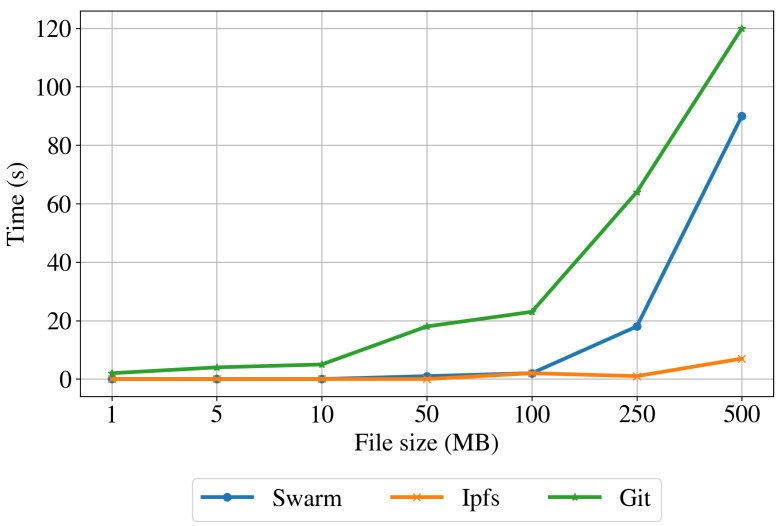
Average download times for different file sizes for the Swarm, IPFS, and Git storage.

Dataset download time. To assess the performance of the proposed architecture in retrieving the data, we measured the query and retrieval times of the datasets [29] when downloading each file approximately 30 min after the upload. As a reference to centralized infrastructure, we download the test file from the Git repository. To this end, we made a pull from the command line, using a pair of register keys as the authentication method. Table 2 shows the minimum, maximum, average, and standard deviation of the download time for Swarm, IPFS, and Git. The table also includes the average peers connected to each node and the average ping time of the Git repository. The downloading results for the three implementations are also shown in Figure 9. For the centralized implementation, the download time shows an exponential increase with the increase of the file size. The download time for both distributed implementations is lower than the Git implementation for all the file sizes. The download time for files up to 100 MB is almost constant for Swarm and IPFS protocols. However, for files larger than 100 MB, the performance degrades slightly for the IPFS and strongly for the Swarm protocol.

**Figure 10:**
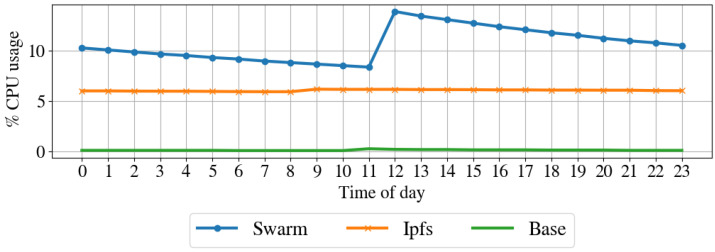
System average CPU usage before and while running the proposed architecture with IPFS and Swarm.

**Figure 11:**
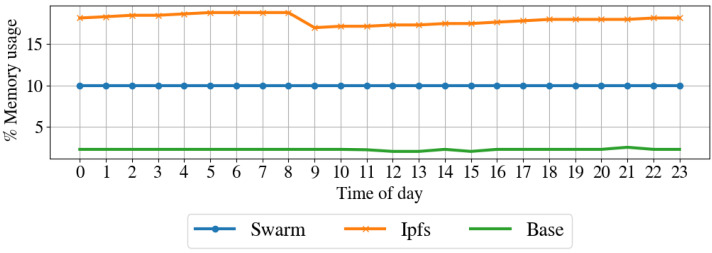
System average memory usage before and while running the proposed architecture with IPFS and Swarm implementation.

**Figure 12:**
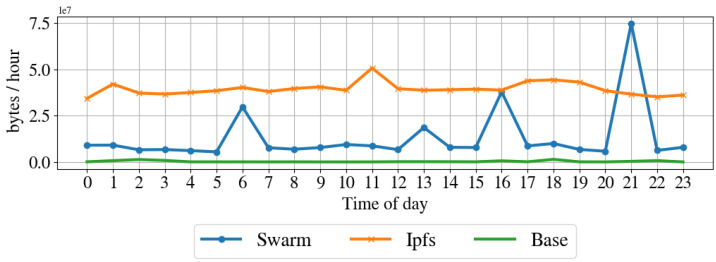
Network average received bytes per hour before and while running the proposed architecture with IPFS and Swarm.

**Figure 13:**
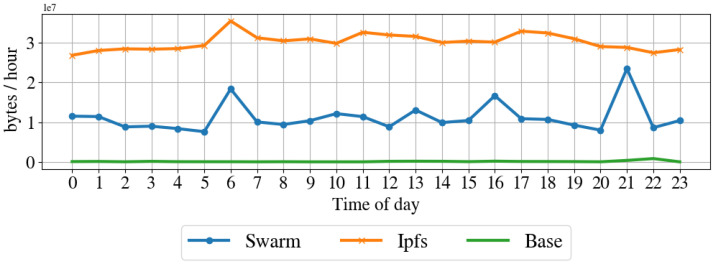
Network average transmitted bytes per hour before and while running the proposed architecture with IPFS and Swarm.

Resources impact on the node. To understand the impact of the proposed architecture in terms of resources for the node, we profile the computer in regular operation by taking a snap-shop of the system resource usage every hour for one week. Then, we performed the same profile while running the node client for IPFS and Swarm. Table 3 shows the average hourly sent and received packets, sent and received bytes, CPU usage, and disk usage for the regular operation, IPFS operation, and Swarm operation modes. Figure 10 shows the CPU usage over one day, Figure 11 illustrates the memory usage over the day, Figure 12 shows the received bytes per hour over one day, and Figure 13 shows the transmitted bytes per hour over one day. The CPU usage of the IPFS implementation and the baseline are constant over the day, even if the IPFS implementation has a larger CPU average usage than the baseline. The Swarm implementation’s CPU usage is average larger than that of the IPFS implementation and varies significantly over the day. The memory usage of the Swarm implementation and the baseline is constant over the day, although the Swarm implementation has a larger memory footprint than the baseline. The memory footprint of the IPFS implementation slightly varies during the day and is on average higher than the Swarm implementation and the baseline. Transmitted and received bytes per hour are on average higher for the IPFS implementation than for the Swarm implementation. The IPFS implementation shows an almost constant behavior over time, while the Swarm implementation has several high peaks of received and transmitted data. To summarize, the Swarm protocol requires less memory than IPSF (see Figure 11) but has a higher CPU usage (see Figure 10). IPFS, on average, has a larger received and transmitted bytes per hour than Swarm (see Figure 12).

#### 4.2.2. Performance of the Blockchain

To evaluate smart contract design, we measure the cost in terms of GAS of each transaction as the metric for comparing different system operations. The GAS allows the comparison of different blockchain-based applications and is directly related to scalability, performance, and monetary costs [36,44]. We also provided transaction cost and transaction processing time as additional metrics.

Transaction costs. Each transaction has an infrastructure cost on public blockchain networks, i.e., a transaction fee. That cost covers the reward of the miners. On Ethereum, a transaction needs gas, the unit of measure of the computations and storage required by a transaction. For instance, a cryptocurrency transfer typically costs 21,000 gas units. More complex transactions have a higher cost as they require more computation and storage. From the user perspective, gas cost translates into monetary cost using a gas price. The gas price is typically expressed in Gwei, defined as $10^{-9}$ of an Ether, the Ethereum cryptocurrency. Higher gas prices provide faster transaction times, giving a higher incentive for miners. The transaction processing time is influenced by several other factors, such as the number of transactions and active peers, that are beyond the scope of this paper. Here, we adopted a gas price of 10 Gwei, the value recommended by software wallets (e.g., metamask) for an average transaction processing time. Finally, to represent this value as a monetary cost, we consider a conversion rate of 1 Ether = USD 300, considering the average ETH price during 2019. Table 4 shows the average size (in bytes) of the metadata and the resulting transaction, gas cost, and monetary cost (in USD) for each of the seven transactions. The ‘Bootstrap’ and ‘Create’ transactions are the most expensive in terms of gas and cost, as the cost in USD equals 11.83 and 3.42, respectively. In terms of size, ‘Create’ and ‘Update’ transactions are the most expensive transactions, occupying 509 and 236 bytes, respectively. ‘Search’ and ‘Detail’ transactions have no gas cost as they require querying the local version of the blockchain.

**Figure 14:**
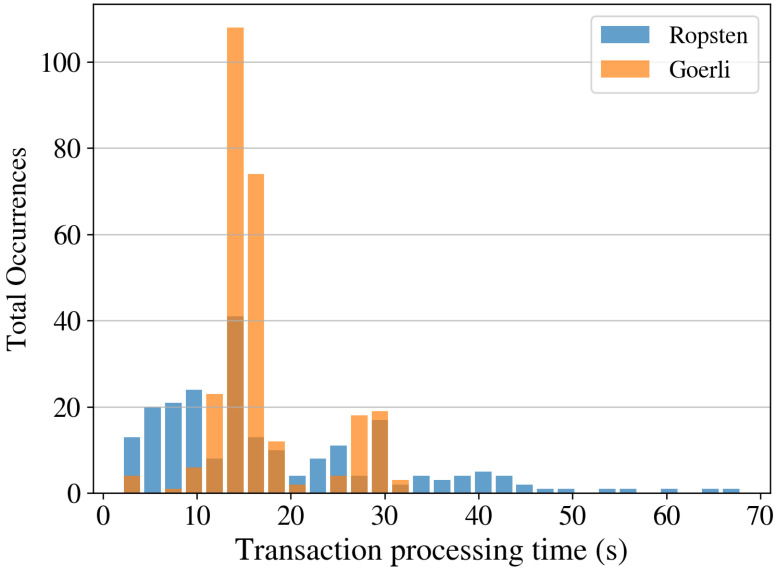
Transaction processing time for Ropsten and Goerli networks.

Transaction processing times. To measure the processing time, we created 36 instances of each transaction type (see Section 3). We send all the transactions approximately 15 min apart to have different network conditions. Table 5 shows the minimum, maximum, mean, and standard deviation of the processing time in seconds for ten instances using a gas price of 10 Gwei for both PoW (Ropsten) and PoS (Goerli) networks. Figure 14 shows the histogram of the processing times for all types of transactions on both PoW and PoS networks. In the Ropsten network with $\mu_{R}=18.35$ and $\sigma_{R}=12.39$, the transactions were, on average, executed in less than 20 s with a maximum time of 68 s. On the other hand, in the Goerli network with $\mu_{G}=16.69$ and $\sigma_{G}=5.8$, the transaction processing time was never larger than 32 s and, on average, around 15–18 s.

#### 4.2.3. Analysis of the Architecture Scalability

We perform several experiments to assess the architecture scalability by significantly changing the number of actors and shared files. We also compared the proposed architecture with three other designs based on current literature. This is to assess the goodness of different smart contract designs quantitatively. The architectures considered in the analysis are the following.

(A1) This architecture is based on [29] and has two smart contracts: DataOwner and DataSharing. A DataOwner smart contract is created for each actor sharing a dataset and contains all the information on the actor. This is far from our proposal, where we only use the cryptographic key of the authors (saved in the catalog smart contract). The data-sharing contract is similar to the Dataset smart contract in our proposal. Each time a file is uploaded, a tuple composed of a KeywordIndex, a transaction id, and an encryption key is added to the data-sharing contract through a function called addIndex. Each time the users search for a file, this contract requires a transaction that modifies the contract, thus imposing a fee for each search. Unlike our proposal, this architecture does not consider dataset scoring, nor does user rewards, does not have an index, and uses an additional contract for each user.
(A2) This architecture is a naive approach, similar to that in [16], where one smart contract is created for each author and shared file. Based on the ERC-20 standard, a controller works as a token provider for rewarding the users.
(A3) This architecture is a simplified version of the proposed one and lacks the rewarding and scoring functionalities. This is done to match the functionalities of architecture A1 and make a more straightforward comparison.
(A4) This architecture corresponds to the proposed one and includes the tracking, scoring, and rewards functionalities.

**Figure 15:**
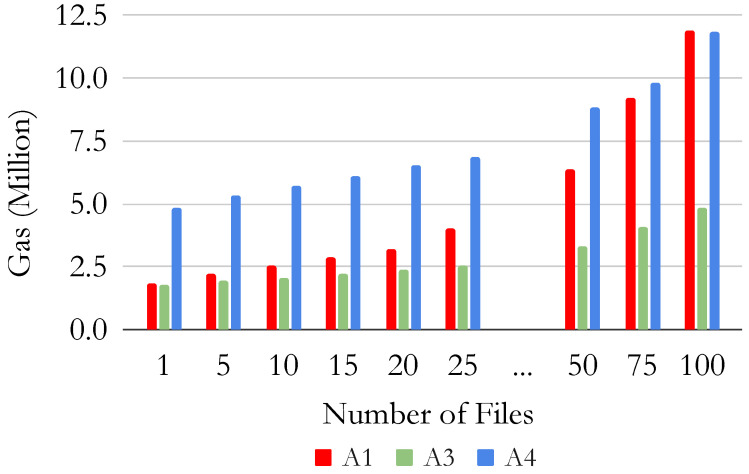
Total gas usage for creating and searching different number of files for Architectures 1, 3, and 4.

Table 6 presents the gas usage of the four implementations for the different functionalities to share different numbers of files. Figure 15 shows the total gas sum of all the operations required to share different numbers of files (i.e., create the controller, the dataset, the user, and then add the files). In this Figure, A2 is not shown since it has the largest gas usage by several orders of magnitudes, and thus makes the comparison with other architectures harder.

The naive approach A2 significantly differs from the other architectures (A1, A3, A4) in terms of GAS since it requires several transactions for sharing and searching datasets. This highlights the goodness of our smart contract design. When sharing one single file, A1 performs better than the other architecture, as it does not require the creation of an additional catalog or controller contract. However, as the number of files increases, the proposed simplified architecture (A3) requires less gas for sharing the same number of files. Furthermore, when sharing more than 100 files, the proposed architecture A4 requires less gas than A1. Moreover, the cost for retrieving a dataset is lower in A3 and A4 than in A1, as A1 has an associated cost when searching a shared file. However, this cost is not present in our architecture as we do not modify the blockchain status and the smart contract when querying a dataset. Furthermore, the cost for adding a user is lower in our proposed architectures A3 and A4 than in A1 as we do not create a new smart contract for each user, but we store only the cryptographic key in the catalog smart contract. Therefore, our proposal presents better scalability when adding more datasets, files, and users, given the careful design of smart contracts. The results show that the performance of the proposed architecture A4 is higher than the architecture proposed in the literature in A1 and A2 when scaling to a large number of actors and shared datasets. Note that the proposed architecture has more functionalities than those in the literature, including the scores for the datasets and rewards for the users.

#### 4.2.4. Thread Model and Security Analysis

Given the exposure of DeBlock to unknown actors sharing datasets in a decentralized way, it becomes susceptible to multiple security attacks, which may impact the system’s trustworthiness, as well as data accessibility and availability [45]. This section discusses the threat model for DeBlock and the related mitigation strategies. Based on current literature on blockchain-based applications security [30,45,46] we considered the following attacks and their corresponding mitigation strategies:

Sybil and Distributed Denial of Service (DDoS) attack on the network. In a Sybil attack, an actor creates multiple identities (i.e., fake users) to manipulate the system in its favor. In a DDoS attack, several actors send simultaneous requests to collapse the system’s availability for accessing the data and metadata. As a countermeasure, our proposal uses a public blockchain network, which highly reduces the possibilities of these attacks [47]. In a public network, the consensus mechanism and the number of honest nodes force the attackers to spend many resources (e.g., energy and computing power) for each attack, greatly reducing their occurrences [45,47].

Smart Contract vulnerabilities. Smart contracts are currently the weakest point of blockchain-based applications [46], as they introduce software vulnerabilities, including transaction order management, reentrancy, integer overflows, and denial of service [45,48]. Our architecture uses a simple and scalable software architecture based on two smart contracts adopting industry-approved libraries. Using these libraries introduces software patterns and best practices to lessen the impact of possible threats. Furthermore, reusing audited code significantly reduces the attack surface of the smart contracts [36], and thus, the DeBlock architecture.

Dataset availability. Dataset availability is related to the presence and accessibility of the datasets and depends on their storage location. Therefore, any attack on the storage could prevent data access for the system’s actors without redundancy [45]. Furthermore, datasets could be lost forever if the storage fails and the data are not replicated elsewhere [30]. In our proposal, the decentralized storage and the blockchain increase the dataset availability as they provide redundancy by replicating the data in more than one network peer [13,17]. For more detail, we refer to the literature [13,17,30] and the references within.

Dataset integrity. This threat is related to modifying the data stored on the blockchain or the storage network. However, blockchains are considered immutable, as the information stored in public blockchains cannot be modified without an extremely significant resource consumption [46]. Similarly, the information on the distributed storage is hardly modifiable as it is replicated and includes cryptographic safeguards [30]. However, in the modification case, the cryptographic safeguards (i.e., a hash) provide information to verify the dataset integrity [13]. If the cryptographic hash is unavailable, an attacker could modify the dataset without the actor noticing [30]. In our architecture, each file uploaded to the DFS is uniquely identified by its cryptographic hash computed automatically. Further, the hash is stored in the blockchain, where it can not be deleted or tampered with, and it is easily accessible to check the integrity of the dataset without revealing its content [17].

Dataset privacy. By default, the public network exposes all the information to all the participants in the system [47]. Even if openness and transparency help improve trust among actors, disclosing the content may lead to privacy leaks, resulting in data misuse for certain datasets [46]. Our architecture supports state-of-the-art encryption techniques, such as ABE [29] or ZKP [15], that can be applied to the dataset. In this way, Deblock provides modularity to share the dataset openly or use complex encryption-based schemes to guarantee the privacy of the dataset content. This approach is aligned with current literature for preserving privacy on blockchain-based applications [15,30,45].

## 5. Conclusions and Future Works

This paper presents a fully decentralized architecture called DeBlock that integrates a public blockchain and a DFS network with an efficient smart contracts design. The public blockchain stores the metadata and allows unknown actors to interact in a trustless way. The DFS stores the encrypted dataset by splitting it among several peers linked to the blockchain through the decentralized interface. Moreover, we propose an innovative and efficient smart contract design that allows the system to scale easily in terms of actor numbers and shared datasets. The system performance is evaluated by implementing a PoC, considering Ethereum as a permissionless blockchain, and two DFS implementations, i.e., IPFS and Swarm. The results showed that the proposed architecture enables the trusted interaction of unknown actors and the storage and update tracking of the datasets. The permissionless blockchain is viable in terms of monetary costs and performance for dataset sharing, allowing trusted interactions between unknown actors. Moreover, the blockchain guarantees data integrity and access to all the actors in the system. Additionally, it provides a tamper-proof history of the dataset, with full traceability of the updates. Considering two different DFS implementations, the architecture’s performance has good results in terms of time, cost, and impact on the node resource. Compared to a centralized storing system (git version control), both DFS implementations require less time for dataset upload and download. IPFS provides a faster upload and download than Swarm at the cost of splitting the datasets into fewer pieces and sending them to closer nodes. Swarm requires more time as it splits the file into more pieces stored in more and farther nodes. Although this strategy increases the time response, it provides a higher level of decentralization and dataset availability. Regarding the impact on the node resources, Swarm requires less memory than the IPSF but has a higher CPU usage. On average, IPFS receives and transmits more bytes per hour than Swarm. Considering the results, the choice of the DFS implementation depends on the requirement of a specific use case. Finally, to evaluate the smart contract design’s goodness, we compared the proposed architecture’s performance with those in the literature. The results show that the proposed architecture is more efficient and scalable in terms of actors and datasets. Furthermore, as the number of actors and files increases, our implementation requires less gas and time while providing more benefits (including the possibility of scoring the dataset and rewards for the data owners) than the literature architecture.

In future works, we plan to evaluate the time response of read-only transactions and the impact of different encryption methods on the data in terms of computational resources, time, and gas usage. We also plan to explore how to incentive data sharing, including developing more attractive rewarding mechanisms.

## Figures and Tables

**Table 1:** Upload times expressed in seconds for the Swarm, IPFS, and Git storage.

File Size	Swarm				IPFS				Git			
	Min	Max	Avg	σ	Min	Max	Avg	σ	Min	Max	Avg	σ
1 MB	1	72	12	16.11	<1	1	<1	0.47	4	6	4	0.75
5 MB	1	45	11	9.89	<1	1	<1	0.44	5	8	6	1.02
10 MB	1	40	10	8.42	<1	1	<1	0.49	6	7	6	0.49
50 MB	9	67	13	14.26	1	2	1	0.25	16	17	16	0.4
100 MB	7	98	40	30.49	1	5	2	0.91	29	30	29	0.49
250 MB	22	522	105	120.03	8	18	9	2.36	82	85	83	1.36
500 MB	58	741	194	155.79	8	18	9	2.36	210	218	214	3.12
	Avg. peers: 29				Avg. peers: 164				Ping time: 23 ms			
	σ = 3.35				σ = 63.54				σ = 0.55			

**Table 2:** Download times expressed in seconds for the Swarm, IPFS, and Git storage.

File Size	Swarm				IPFS				Git			
	Min	Max	Avg	σ	Min	Max	Avg	σ	Min	Max	Avg	σ
1 MB	<1	1.0	<1	0.22	<1	1.0	<1	0.24	2	3	2	0.49
5 MB	<1	1.0	<1	0.22	<1	1.0	<1	0.22	3	20	5	5.4
10 MB	<1	1.0	<1	0.22	<1	1.0	<1	0.25	2	9	5	2.42
50 MB	<1	2.0	1	0.46	<1	1.0	<1	0.25	12	43	18	12.12
100 MB	1	4	2	0.7	<1	28.0	2	6.77	5	52	23	17.55
250 MB	4	205	18	49.9	<1	2.0	1	0.32	53	87	64	13.63
500 MB	8	1218	90	301.38	1	86	7	21.03	104	156	120	18.29
	Avg. peers: 44				Avg. peers: 933				Ping time: 42 ms			
	σ= 11.32				σ = 79.99				σ = 25.93			

**Table 3:** Resource impact on the node in terms of CPU and memory usage, and transmitted and received bit per hour before (Base Line) and while running the proposed architecture with IPFS and Swarm.

Metric	Base Line	Swarm		IPFS	
	Avg	Avg	δ	Avg	δ
CPU Usage (%)	0.20	10.62	10.42%	6.04	5.84%
Memory Usage (%)	3.00	10.04	7.04%	18.01	15.01%
Network Tx (b/h)	82,138.31	11,202,555.10	11,120,416.79%	31,273,935.23	31,191,796.92%
Network Rx (b/h)	619,528.38	13,026,146.52	12,406,618.15%	38,839,748.92	38,220,220.54%

**Table 4:** Transaction cost (gas usage) using different size of metadata.

Transaction		Metadata	Gas
(Type)		(Byte)	(Units)
(0)	Bootstrap	10	3,589,913
(1)	Create	100	1,200,317
		200	1,209,577
		400	1,228,036
		800	1,264,490
		1000	1,284,496
(2)	Update	100	43,299
		200	51,167
		400	66,777
		800	97,690
		1000	113,822
(3)	Authorize	50	77,402
(4)	Reject	42	30,025
(5)	Search	-	0
(6)	Request	14	43,507
(7)	Details	-	0
(8)	Score	4	64,120

**Table 5:** Create transaction processing times in terms of minimum, maximum, average, and variation on a Ropsten and a Goerli network.

Transaction		Ropsten (PoW)				Goerli (PoS)			
		Min	Max	Avg	σ	Min	Max	Avg	σ
(0)	Bootstrap	06	40	19.14	10.74	08	24	17.00	5.92
(1)	Create	04	68	19.03	15.27	02	32	15.30	5.38
(2)	Update	04	48	17.50	10.81	12	30	17.20	5.05
(3)	Authorize	06	50	24.61	11.14	12	30	17.13	7.11
(4)	Reject	08	60	22.83	14.26	10	30	17.40	7.08
(6)	Request	06	42	17.69	10.19	02	32	16.57	5.23
(8)	Score	04	40	13.56	8.82	14	32	17.53	5.72

**Table 6:** Gas usage compared to state of the art with different number of files.

Transaction	Files	Gas			
		A1	A2	A3	A4
Create Controller		0	2,644,822	1,274,321	3,589,913
Create Dataset	-	1,143,873	676,611	1,800,714	4,845,861
Create User	-	529,014	0	0	0
Add User	-	44,349	0	0	0
Add File	1	676,611	908,355	0	0
	5	292,879	3,324,747	122,160	468,504
	10	545,233	6,634,917	275,703	872,125
	15	797,461	9,945,087	428,040	1,275,938
	20	1,049,690	13,255,257	581,044	1,679,751
	25	1,846,893	16,565,427	733,344	1,993,817
	50	3,693,683	33,116277	1,497,788	3,988,850
	75	5,540,986	49,667,127	2,262,296	4,983,755
	100	7,387,110	66,217,977	3,025,972	6,977,892
Search File	1	74,318	0	0	0
	5	274,367	0	0	0
	10	311,548	0	0	0
	15	388,764	0	0	0
	20	473,160	0	0	0
	25	519,833	0	0	0
	50	989,034	0	0	0
	75	1,987,560	0	0	0
	100	2,859,182	0	0	0

## Data Availability

Not applicable.
